# Introducing hyaloid-sparing double capture: a novel technique in cataract surgery—anticipated clinical trial rationale and invitation

**DOI:** 10.3389/fopht.2025.1689724

**Published:** 2026-01-07

**Authors:** Lisa Brothers Arbisser

**Affiliations:** John A. Moran Eye Center, College of Health, University of Utah, Salt Lake City, UT, United States

**Keywords:** anterior hyaloid membrane, Berger’s space, 3-piece lens, zonulopathy, posterior capsulorhexis

## Abstract

The state of the art of in-the-bag cataract surgery, though one of the most successful surgeries in ophthalmology, has significant room for improvement as a one-time, lifetime solution for optical clarity. Though optic capture techniques have been known since the 1990’s they are underutilized and not adequately explored or understood. This perspective not only explains their history and value but also clarifies the anatomy and suggests a proven improvement and a proposed study for another solution: hyaloid sparing double capture, to once again elevate the outcomes and lessen the complications of our routine surgery. Open your mind: hone your skills with this knowledge and help us move forward.

## Consider expanding your skill set because

In state-of-the-art cataract surgery, we implant an intraocular lens (IOL) in the bag after lens removal. Despite being one of the most successful ocular surgeries, it induces blood–aqueous barrier breakdown, disrupts contact inhibition, and initiates a capsular “healing” response. Lens epithelial cells (LECs) proliferate, resulting in Elschnig pearl formation and Soemmering’s ring. Contact between implant materials and the apposition of anterior and posterior capsule LECs causes both fibroblastic and myofibroblastic transformations, resulting in anterior capsule rigidity and contraction. These phenomena often lead to IOL movement resulting in unpredictable effective lens position, IOL decentration, tilt, and anterior capsular rim phimosis with only the zonules to counteract these forces. More rarely but significantly, bag–lens complex subluxation results. Eyes most at risk include those with pseudoexfoliation (PXF), post-vitrectomy, and traumatic cataracts. Uveitic and pediatric eyes are more prone to inflammation and aggressive fibrosis.

## The anterior hyaloid is sacrosanct: NOT the posterior capsule

Historically considered relatively benign, Nd: YAG laser posterior capsulotomy (PC) is increasing in frequency due to the compounding of the posterior capsule’s imperfect clarity with stray light effects interacting with refractive IOL contrast and dysphotopsia issues (1). Of more recent significance, the effective lens position can change following Nd: YAG PC, encouraging early capsulotomy as part of the process associated with the Light Adjustable Lens (RxSight) before lock-in. Because the onset of posterior capsule opacification (PCO) is dependent on technique, IOL material, and design, the incidence and timing of visual axis opacification vary wildly from 3% to 80% in the literature.

Nd: YAG PC (2) universally disrupts the anterior hyaloid membrane (AHM)—the true barrier between anterior and posterior segments. AHM violation is associated with floaters, and more rarely, retinal tears and detachment, increased risk of cystoid macular edema, progression of diabetic retinopathy, and macular neovascularization, presumably due to access of anterior segment cytokines to the posterior segment.

With the passage of time (1), mature fibrotic barrier formation following bag implantation isolates the anterior segment from the posterior segment after cataract surgery, but early postoperative capsulotomy risks creating a one-chambered eye. Unfortunately, the standard of care for pediatric cataract surgery (at least in the USA) involves primary anterior vitrectomy, vitrectorhexis, and IOL in the bag (3). Understandably, there is a higher incidence of problems associated with the communication between the anterior and posterior segments. In adults, anterior segment anatomical changes following laser PC with a more posterior movement of the lens-iris diaphragm may benefit glaucoma patients (4). These changes may prove to be seen with primary optic capture without violation of the hyaloid primarily. Even when none of these consequences occur, although Nd: YAG PC is quick and effective, the patient must experience a second period of visual degradation before the procedure is approved in the non-refractive setting. This adds huge economic cost to the healthcare system and is not always available in a timely manner to those in the developing world.

Fibrosis and phimosis of the lens capsule with in-the-bag implantation contribute to an ever-increasing incidence of a more vision-threatening sequela of standard surgery: late bag–lens complex subluxation, most common in patients with preoperative or intraoperative zonular pathology, is not always recognizable. On average, it takes 5–10 years for clinically significant subluxation to occur, requiring intervention. Today, patients undergo cataract surgery at earlier ages, and dysfunctional lens syndrome is increasingly treated with refractive lens exchange. Referral centers often treat several subluxations per week. These surgically challenging eyes are even encouraging consideration of a new subspecialty, “middle segment surgery”, to handle the complexities required for optimal results.

## Optic capture: haptics and optics on either side of a stable membrane

All of the above offer compelling reasons to advance the art of cataract surgery by employing membrane capture techniques. Although Weidle (5) was the first to publish intentional intraoperative manipulation of a thickened posterior capsule during extracapsular cataract extraction, and Stegman captured the optic behind the posterior capsule through a slit opening, the seminal discovery that makes all options feasible is that of continuous curvilinear capsulorhexis (CCC), which is credited to Gimbel and Neuhann in 1990. CCC makes it possible to secure the IOL position for uncomplicated surgery and intraoperative complications; it facilitates anterior capture, reverse capture, posterior optic capture, fibrotic membrane capture, and haptic tuck techniques, which are all established means of attaining the best outcomes (6).

This author began learning planned primary posterior capsulorhexis and using primary posterior optic capture (PPOC) for any patient who could not sit for a Nd: YAG PC, during traumatic situations, and for pediatric cataract implantation in 2007 after learning of Menapace’s definitive prospective intra-patient, randomized controlled studies (7). He masterfully proved non-inferiority with complete elimination of PCO, superior vision due to less stray light, stable effective lens position, and preservation of the two-chambered eye. There was no increase in intraocular inflammation, endothelial cell or corneal clarity, cystoid macular edema (CME), early ocular hypertension, or retinal detachment (8). We can logically presume reduced risk of retinal and glaucoma problems associated with anterior long-term hyaloid violation beyond his study protocol. He makes a compelling argument for posterior optic “buttonholing” or capture (POC) as the routine cataract surgery technique of the future for all ages in this Frontier’s edition and his many publications. There is a steep (short) learning curve, and complications are few. If we size a stable anterior CCC to be capturable, we can always rescue a complication with reverse optic capture or anterior capture.

Although a double-capture IOL position in pediatric cataract was previously described by Gimbel and DeBroff, it was always with coincident primary anterior vitrectomy and posterior vitrectorhexis. Vasavada and others have shown it can be otherwise with POC sparing the AHM, providing a long-term, clear visual axis (9). DeBroff, well-practiced in standard technique, was motivated to change to a hyaloid-sparing method. Perhaps the 30% incidence of postoperative pediatric glaucoma may decline with an intact AHM. Theoretically, the access of oxygen to the developing trabecular meshwork may play a role (although the causes are poorly understood and multifactorial).

We are indebted to another master surgeon and teacher of the little-understood retrolenticular anatomy, Tassignon, with her unique IOL design (forerunner of my concept), the Bag-in-the-Lens (BIL) (Morcher) (not Federal Drug Administration (FDA) approved) (10). BIL has a collar button-like 360-degree groove into which perfectly centered and sized anterior and posterior rhexis edges are inserted (a more advanced skill than PPOC) (11). It provides longstanding safety and spectacular pupil-edge to pupil-edge clarity at all ages over time, with a non-visually significant Soemmering’s ring identified by Werner in a post-mortem specimen, a hopeful sign regarding dead bag prevention (12, 13). The BIL is, however, totally zonule dependent, sometimes requiring bean-shaped capsular tension rings (CTRs) to anchor it to the sulcus for centration and stability.

Experience teaches that (unlike in post-Nd: YAG patients) lens exchange is uncomplicated in POC. The captured incorrect IOL is easily disenclavated from its capture, while the AHM remains intact and the new lens is captured.

## Zonulopathy

First recommended in 2003 by Safran in a non-peer-reviewed journal and independently intuited, presented, and practiced by me, I found that primary anterior capture of a three-piece IOL with haptics in the sulcus and CTR in the bag for significant zonulopathy led to a very stable IOL without pseudophacodonesis, presumably due to the blockage of phimosis by the anterior captured optic. Anterior capsule Nd: YAG relaxing incisions were unnecessary. Unfortunately, due to the loss of any sandwich effect, these eyes uniformly require early Nd: YAG posterior capsulotomy for acceptable visual outcomes.

## HSDC: haptics in the sulcus, optic In Berger’s space

In 2014, although yet unpublished, I presented the Wolfe lecture at the University of Iowa, which is now agreed (even by Gimbel) to be an original idea of combining the technique of PPOC with sulcus placement and anterior capture following a primary posterior continuous capsulorhexis, hence hyaloid-sparing double capture (HSDC).

Results of HSDC cases performed by Oetting at the University of Iowa (yet unpublished) demonstrated technical feasibility and stable fixation 10 years postoperatively.

I hypothesize that POC, for certain, and HSDC (to be determined) will result in the most stable lens possible at the plane of the bag (although the nomogram remains to be calculated, and most are currently adding +0.5D on average), with zero PCO and potentially flexible capsules equator to equator at any age. Although a Soemmering’s ring may form like in BIL, it would be confined to the equatorial space by the optic capture, which seals the anterior and posterior capsule edges together except at the optic–haptic junction, sequestering antigen. Standard three-piece IOLs should always be centered based on support in the sulcus and mostly by the centration of the anterior rhexis into which it is captured. This may also reduce the chances of uveitis-glaucoma-hyphema syndrome (UGH) syndrome, as there is no pseudophacodonesis. Unlike today’s standard lens in-the-bag operation, the CCC is stented by the optic, excluding phimosis, and thus, theoretically, late bag–lens subluxations are impacted. Since there is virtually no touch of IOL to lens epithelial cells, metaplasia will not be stimulated, and the bag should remain flexible. As another bonus, an IOL implanted with the capture technique requires no square edge, reducing dysphotopsia, and could be used for all techniques and many complications. When intra-operative complications prevent the use of the bag to implant a stable single-piece IOL, the patient (and surgeon) often must relinquish the benefits of advanced refractive technology—at least in the USA. Additionally, a captured toric lens can never rotate. Various forms of optic capture can save the patient from serious consequences when the bag is not viable for a single-piece IOL. This argues for mastering capture techniques and a plea to the industry to offer newer refractive three-piece IOL designs useful for any form of effective capture, in-the-bag use, and even scleral fixation (the topic for another paper).

Thus, HSDC secures the lens, removes the posterior capsule as a scaffold for LEC proliferation (keeping the visual axis clear), and spares the AHM for life, maintaining the two-chambered eye with all its known and potential advantages. Centration is sulcus-supported and dependent on anterior capsule centration. If all zonules were lost, there would technically be no sulcus, unlikely because the incidence of spontaneous natural crystalline lens subluxation is vanishingly small.

## Anatomy

The posterior capsule is 4–6 μm thick and more elastic than the 14–16-μm anterior capsule. The capsular bag is bordered by the zonular network’s interdigitated fibers around its equator for 360 degrees. The posterior zonular attachments of Wieger’s ligament define Berger’s space with a diameter of 8–9 mm centrally, with the space of Petit peripherally. The posterior capsule (PC) is anterior, and the AHM is posterior. Berger’s may be real or potential in the natural state, most often requiring definition with a viscoelastic ophthalmic viscosurgical device (OVD) to adequately separate the anterior vitreous face from the posterior capsule for safe manipulation. On femtolaser optical coherence tomography (OCT), pioneered by Dick, one can see the necessary 400 μm of space between the two in at least 72% of all eyes and 100% of eyes with axial length over 25 mm, immediately after phacoemulsification surgery. An OVD is unnecessary to permit femtolaser posterior capsulotomy. He pioneered the femto posterior capsulotomy (without capture) (off-label), even providing a nomogram for posterior capsulotomy sizing in pediatric cataracts based on age (14, 15).

Intraoperative and modern anterior segment optical coherence tomography (AS-OCT) office models are now capable of imaging the retrolenticular space when desired, but we owe much of our modern understanding of the anatomy to the work of Weidel, Worst, and Tassignon (16, 17).

## Surgical technique

HSDC consists of three key steps:

1. Dual capsulorhexes
2. A three-piece IOL is introduced into the sulcus
3. Double capture of the optic into Berger’s space

## Details

Following standard lens removal through a 5-mm (capturable) centered anterior capsulorhexis, the posterior capsule and anterior capsule rim are polished according to the surgeon’s preferred method. A cohesive OVD is placed in the sulcus rather than the bag to flatten the anterior capsule rim and posterior capsules together into one plane. Once the posterior capsule is planar, on high magnification with a well-coated or wetted corneal surface facilitating visualization, a 30-gauge bevel-up needle on a tuberculin (TB) syringe handle is used to create a crease in the PC, thereby lifting it to puncture the central posterior capsule, lifting it away from the AHM (in case Berger’s space is only potential), and pushing forward and sideways as one would with anterior CCC to start a curvilinear flap. Although this is a similar maneuver to anterior CCC, a cystotome should not be used, as the AHM may be closely applied to the posterior capsule, and both would be inadvertently opened by the downward-facing barb. Once the flap is initiated, a cohesive OVD on its native cannula (air bubble excluded by filling the OVD cannula hub with balanced salt solution (BSS) before attaching it) is instilled under the posterior capsule to define Berger’s space, pushing the hyaloid posteriorly out of harm’s way to its border at Wieger’s ligament. Although unnecessary, if one has intraoperative OCT, that diaphanous membrane can be seen floating immediately backward and staying put. If the OVD dollop does not become circular out at approximately 8-mm circumference, it is likely that Wieger’s ligament is not intact, which is not a problem. The other possibility is that the posterior capsule and anterior hyaloid membrane are not separated. Note there is a malformation of Berger’s space in some posterior congenital cataracts. Even in adults, there is no absolute guarantee that in patients who did not have a detached AHM before surgery, or during surgery, we will always be in the right place, in which case the OVD will only compartmentalize the anterior vitreous (18). In such cases, the optic capture seals the two-chambered eye without known sequelae. Menapace prefers to make at least one-quarter posterior continuous curvilinear capsulorhexis (PCCC) before injecting the OVD to effectively avoid this occurrence. Judgement is gained with practice to decide when OVD is sufficient. The leading edge of the partial circular tear is then grasped using Utrata (or similar) forceps to complete the CCC. Unlike the anterior capsule, the PC behaves more like elastic pediatric tissue, so a centripetal vector of force must be applied to properly direct the tear, re-grasping more frequently to correct the vector force. The anterior rhexis is used, like a cookie-cutter template, to judge the size, shape, and location of the posterior rhexis, which is intended to be slightly smaller. As seen in standard pediatric anterior continuous curvilinear capsulorhexis (ACCC), the last few millimeters of the rhexis require a Gimbel-described “Little rescue maneuver”, pulling the flap backward to the direction of the tear to complete the circle without it spiraling and enlarging. A curvilinear rhexis finishing outside where it began to achieve continuity is executed (19).

Once completed, the detached capsular flap is removed. This maneuver happens without any hint of posterior pressure in virtually every case. There is little or no tendency for it to “run out” since there is no convexity of the capsule with posterior pressure, unlike the anterior rhexis with the lens body intact. A CTR can even be implanted as needed once the PCCC is begun if zonules are very lax without extending the tear.

Next, for HSDC, rather than inflating the capsular fornix to implant the IOL (as with standard in-the-bag or posterior buttonhole technique), the two capsules are left in apposition. This facilitates first the leading haptic and then the entire body and trailing haptic of the IOL to be injected into the sulcus. Once in place, the optic is captured into Berger’s space (leaving the haptics in the sulcus) by pressing on the optic edge 90 degrees away from the optic–haptic junction on both sides, causing the typical football or cat-eye appearance of the capsule edges over the optic and under the haptic junctions, confirming capture. Unlike anterior rhexis capture, due to the posterior capsule’s thin and elastic nature, the optic may not just “pop” under the capsule edge but may sometimes require being “walked” under the PCCC edge by pressing gently on the edge 90 degrees away from each haptic junction for complete capture. With the optic in Berger’s space safely captured, the OVD can now be removed by coaxial or bimanual irrigation and aspiration from the anterior chamber in the usual manner. The posterior chamber and the posterior segment are now hermetically sealed by the optic, and no attempt is made to remove the OVD behind the lens. Any OVD left in this space has no access to the trabecular meshwork and has been proven (by Menapace) not to cause a pressure rise. It is important not to allow the anterior chamber to collapse, potentially un-capturing the IOL and possibly allowing vitreous prolapse. As should be conducted even in the standard technique (as shown by Osher), irrigation of the incision prior to OVD removal, so it will close upon removal of the irrigation and aspiration (I/A) tip, is advised. In the first few cases, consider irrigation of diluted Triesence into the anterior chamber at the end of the case just to confirm no vitreous prolapse is present as a final maneuver. These eyes will, however, not require the anti-inflammatory effect of this drug and will prove surprisingly quiet postoperatively compared to those that underwent the standard technique.

## Proposed study

We propose consideration of a randomized clinical trial to compare HSDC with standard in-the-bag IOL implantation in eyes that are most at risk for late subluxation and dislocation due to PXF. A registration study with multiple sites nationally and internationally is being considered as the protocol is being standardized.

DeBroff (Yale) and Chaya (Utah) are architects of this study, along with this author and other advisors worldwide. Zonular pathology will be scored in a standardized fashion preoperatively and intraoperatively for each study eye. For the purposes of potential future rescue of bag–lens complex subluxation, a capsular tension ring will be placed in the bag in both the randomized study and control eyes (routine in-the-bag surgery) during surgery (technique to be elucidated). Any three-piece IOL of the investigator’s choosing could be employed. Intraocular lens bag stability will be evaluated postoperatively based on change in anterior chamber depth (primary endpoint), centration, tilt, pseudophacodonesis, phimosis, and visual axis obscuration for a minimum of 2 years and, hopefully, beyond. Lens complex stability will be most efficiently and reliably documented with a custom module on the iTrace eye scanner (Tracey Technology) as well as OCT. The actual protocol is nearing final development. True subluxation, most commonly presenting 5–10 years postoperatively, will hopefully be recorded as well. Intraoperative complications will be noted, and those eyes will be excluded from the study. This author will offer training and coaching. Please contact us if you are interested.

## Summary

PPOC is a proven technique that this author believes should be adopted by all cataract surgeons as an advancement in the art of cataract surgery worldwide.

HSDC integrates the known benefits of POC’s elimination of secondary visual obscuration with anterior capsule capture’s blocking of phimosis. It avoids AHM disruption to maintain the patient’s quality of life. If validated in a prospective randomized trial as planned, it may represent an important advancement in cataract surgery, particularly for high-risk groups such as uveitic patients, as well as those with zonulopathy, including PXF, post-vitrectomy, and iatrogenic and ocular trauma. There is evidence that healthy cells can live in the bag equator. More theoretically, HSDC, if used routinely, may impact the mysterious entity of dead bag syndrome, for which there is no explanation or theoretical protection. As we operate on cataract patients at younger ages and address dysfunctional lens syndrome with refractive lens exchange, without new approaches to our techniques, long-term complications for the world’s most common and effective ocular surgery may only increase. The learning curve is short and manageable. It is more of a mindset than a technical challenge. This author is dedicated to teaching the few extra steps beyond that of the anterior capsulorhexis skills every cataract surgeon has already mastered (as Menapace taught me) for those interested in performing our proposed study.

## Conclusion

Posterior optic capture and the Bag-in-the-Lens approach both demonstrate that PCO prevention is achievable without added morbidity. I have reviewed the role of primary posterior capsulorhexis in cataract surgery, emphasizing its safety and utility in both pediatric and adult populations. HSDC represents a promising new technique in cataract surgery that merges the best features of posterior capsulorhexis, posterior optic capture, and anterior capsule fixation. If validated in clinical trials, HSDC may redefine the standard of care for both adult and pediatric cataract surgery, particularly in eyes predisposed to fibrosis or zonular weakness.

I look forward to a day when secondary visual obscuration is eliminated, effective lens position is predictable and stable, and late bag–lens complex subluxation is substantially reduced if not eliminated. When I need cataract surgery, my optic will be in Berger’s space. Thank you for your consideration. Open your minds and hone your skills.

## Data Availability

The original contributions presented in the study are included in the article/supplementary material. Further inquiries can be directed to the corresponding author.
